# Informed Consent in AI-Augmented Dentistry and Dental Research: A Scoping Review

**DOI:** 10.3390/dj14060320

**Published:** 2026-05-25

**Authors:** Tamara Mihut, Corina Marilena Cristache, Luminita Oancea, Victor Nimigean

**Affiliations:** 1 Doctoral School, “Carol Davila” University of Medicine and Pharmacy, 37 Dionisie Lupu Street, 020021 Bucharest, Romania; tamara.mihut@drd.umfcd.ro; 2 Department of Dental Techniques, “Carol Davila” University of Medicine and Pharmacy, 8, Eroii Sanitari Blvd., 050474 Bucharest, Romania; 3 Department of Prosthetic Dentistry, Faculty of Dentistry, “Carol Davila” University of Medicine and Pharmacy, 19 Plevnei Ave., 010221 Bucharest, Romania; luminita.oancea@umfcd.ro; 4 Romanian College of Dentists, 9, Lainici Street, 012251 Bucharest, Romania; 5 Department of Anatomy, Faculty of Dentistry, “Carol Davila” University of Medicine and Pharmacy, 8, Eroii Sanitari Blvd., 050474 Bucharest, Romania; victor.nimigean@umfcd.ro

**Keywords:** artificial intelligence, informed consent, dentistry, ethics, patient autonomy, decision support systems, clinical, research ethics, data privacy

## Abstract

**Background/Objectives:** Artificial intelligence (AI) is increasingly used in dental diagnostics, treatment planning, documentation, and research. However, there is limited synthesis of how informed consent should be understood and operationalized in AI-augmented dentistry. This scoping review aimed to map the existing literature on informed consent in AI-assisted dental care and dental research, identify conceptual and practical gaps, and synthesize key domains relevant to ethically robust implementation. **Methods:** This review was conducted in accordance with PRISMA-ScR and the review question was developed using the Population–Concept–Context framework. Searches were performed in PubMed, Web of Science, and ClinicalKey, supplemented by Google Scholar and reference list screening. English-language sources published between January 2015 and January 2026 were considered if they addressed informed consent, patient information, autonomy, transparency, accountability, or governance in relation to AI use in dentistry or dental research. **Results:** Of 2624 records identified, 30 sources were included. The reviewed literature consistently emphasized the importance of disclosing AI involvement, clarifying clinician accountability, communicating uncertainty and bias, distinguishing clinical care from research-related consent, and addressing secondary data use. Most included sources were conceptual, ethical, regulatory, or narrative in nature, with limited empirical evidence on implementation or patient outcomes. **Conclusions:** The available literature suggests that informed consent in AI-augmented dentistry should extend beyond traditional clinician–patient models to explicitly address AI involvement, human oversight, and data governance. Based on recurring themes across the included sources, we propose the ACCOUNT-AI framework as a conceptual synthesis to support future research, policy development, and implementation efforts.

## 1. Introduction

Artificial intelligence (AI) is rapidly transforming clinical practice across medicine and dentistry, with applications spanning diagnostic imaging, clinical decision support (CDS), natural language processing (NLP), automated documentation, electronic record analysis, and patient-facing digital health tools. In dentistry, AI-enhanced systems have demonstrated high diagnostic performance in radiographic interpretation—such as caries detection, periodontal bone loss assessment [1], CBCT segmentation and cephalometric landmark identification [2]—as well as in procedural planning, including implant placement and orthodontic treatment simulation [3,4,5,6,7]. Beyond imaging, the published dental literature increasingly describes AI applications in data-rich clinical and research workflows, including extraction of information from electronic dental records, linked electronic dental record/electronic health record (EDR/EHR) prediction models, oral health management systems, and patient-facing chatbot tools for education and communication [8,9,10,11,12]. In parallel, large language models (LLMs), conversational agents, and other AI-enabled communication tools are increasingly being discussed in relation to clinical documentation, workflow optimization, patient information, and digital health support, while mobile health (mHealth) applications and chatbots may assist with symptom triage, education, and self-management [13,14,15,16].

Despite the accelerating adoption of these technologies, there remains a critical gap in understanding how AI reshapes foundational elements of dental practice—particularly informed consent and the evolving dentist–patient–AI relationship [15,17].

Informed consent is a cornerstone of ethical healthcare practice, rooted in respect for patient autonomy, adequate understanding of risks and benefits, and shared decision-making [18]. Traditional consent frameworks are grounded in a bilateral interaction between clinician and patient, relying on the transparent communication of clinical information and the clinician’s professional judgment [19]. However, the introduction of AI as a third actor—whether visible (e.g., CDS systems, chatbots, AI-generated recommendations) or invisible (e.g., backend imaging algorithms influencing clinician interpretation)—fundamentally challenges this model. While the existing literature in dentistry and medicine has addressed general ethical principles related to AI, such as transparency, accountability, explainability, and fairness [5,20,21,22], and has proposed high-level checklists or guidance for AI-related consent [23], we did not identify, in our scoping of the literature, a prior review focused specifically on informed consent in dentistry through the relational lens of the dentist–patient–AI triad.

An emerging and still underexplored dimension of AI integration in dentistry is the increasing use of generative AI and patient-facing health advisors by patients themselves for symptom interpretation, information seeking, and preliminary treatment-related questions prior to professional consultation. Recent studies evaluating AI chatbots in implant dentistry suggest that patient-facing AI may influence expectations, perceived understanding, and decision-making even before direct clinical encounter [11,24]. Such AI-mediated “preconsultation” use has the potential to reshape patient expectations and autonomy, raising important questions about the validity and ethical robustness of informed consent in AI-augmented dental care.

Moreover, the literature has insufficiently explored how AI alters the structure, meaning, and practical execution of informed consent within routine dental workflows.

A further and increasingly consequential challenge is the growing mismatch between the speed of scientific and technical advancement in AI and the slower evolution of regulatory, legal, and professional governance frameworks. While AI systems are rapidly moving from research prototypes to real-world clinical deployment, regulatory guidance regarding their appropriate use, transparency requirements, liability attribution, and documentation standards remains fragmented, incomplete, or reactive rather than anticipatory [25,26,27]. In dentistry, this challenge becomes particularly relevant as AI expands beyond image interpretation into data extraction, longitudinal record linkage, clinical documentation support, patient communication, and other digital workflow functions, thereby generating consent-relevant questions across the clinical and data lifecycle [8,10].

An additional and often underexplored dimension concerns informed consent for AI research, particularly when clinical data are reused for algorithm development, validation, or continuous learning. A conceptual distinction must be made between consent for the secondary use of patient data in AI research and model training and consent for the use of AI systems in clinical decision-making. Consent for data use—typically embedded within research protocols, institutional review board (IRB) approvals, or privacy notices—does not necessarily ensure that patients understand how their data contribute to AI model development, potential future reuse, data sharing across institutions, or the evolving nature of learning systems. Nor does it imply that patients are aware of, or agree to, downstream clinical deployment of AI tools trained on such data. In dentistry, where imaging datasets, intraoral scans, photographs, and salivary biomarkers or behavioral data, among others, are leveraged for AI research, this distinction becomes particularly salient and ethically consequential [8,9,17]. Recent work using dental NLP and linked EDR/EHR machine learning further illustrates that dental data are being mobilized not only for immediate care, but also for broader analytic, predictive, and secondary use purposes [9].

Conversely, informed consent in the context of AI-augmented clinical care must address the role of AI in diagnosis, risk prediction, treatment planning, documentation, patient communication, and data-supported workflow processes. It must also clarify the limits of AI performance, the presence of uncertainty, and the locus of responsibility for clinical decisions.

Emerging phenomena such as automation bias—where clinicians may over-rely on AI outputs—overtrust, in which patients may attribute excessive authority to algorithmic recommendations, and deskilling, reflecting the potential erosion of clinician expertise through sustained dependence on AI systems, further complicate the ethical landscape [28,29,30]. These dynamics raise critical questions about whether consent that is formally obtained is also ethically meaningful, particularly when AI influences decisions in opaque or indirect ways.

Current ethical and regulatory discussions frequently remain at the level of abstract principles and lack actionable frameworks tailored to dental practice and research contexts. There is a clear need for a conceptual and practical model of informed consent that explicitly incorporates the dentist–patient–AI triad and accounts for the heterogeneity of AI applications in dentistry, including AI-assisted diagnostics, documentation support, patient-facing tools, data-driven clinical workflows, and AI-enabled research or screening initiatives.

Accordingly, this scoping review [31] explores how informed consent processes in dentistry may need to evolve, addressing:

- Explicit communication of AI’s role in clinical reasoning, documentation, and research;
- Clear statements regarding clinician oversight and ultimate decision-making responsibility;
- Differentiation between consent for AI research and consent for AI-assisted clinical care;
- The potential utility of dynamic or tiered consent models reflecting varying degrees of AI involvement.

**Figure 1:**
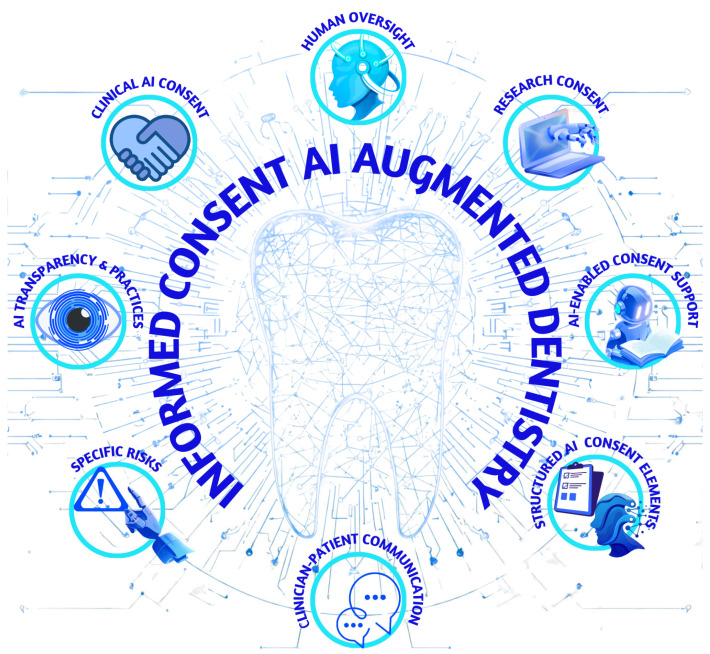
Conceptual framework of informed consent in AI-augmented dentistry, emphasizing that human oversight and clinician–patient communication remain the central ethical and legal anchors, ensuring that artificial intelligence functions as a transparent decision support tool within both clinical care and research contexts rather than as an autonomous decision-maker.

Finally, although preliminary studies suggest that AI-generated consent documents may improve readability and completeness in certain clinical contexts [32,33,34], it remains unclear whether such approaches enhance or undermine patient understanding, trust, and the therapeutic alliance in dental care. This scoping review therefore aims to synthesize existing evidence on informed consent in AI-augmented dentistry and dental research, identify conceptual and practical gaps, and propose a structured framework to support ethically robust implementation of AI in both clinical and research settings. Figure 1 graphically summarizes the overarching aims of this scoping review, illustrating the integrative pathway from evidence synthesis to framework development for informed consent in AI-augmented dentistry.

## 2. Materials and Methods

This scoping review was conducted and reported in accordance with the PRISMA Extension for Scoping Reviews (PRISMA-ScR) [31] (Supplementary Material 1) and was registered with the Open Science Framework (OSF; 10.17605/OSF.IO/U9Y67; accessed on 21 February 2026). The review question was formulated using the Population–Concept–Context (PCC) framework, focusing on dental patients, dental clinicians, and dental research participants (Population); informed consent and ethical, legal, and relational implications of artificial intelligence (Concept); and AI-augmented dental care and dental research settings (Context).

A systematic literature search was performed in PubMed, Web of Science, and ClinicalKey, complemented by supplementary gray literature screening in Google Scholar and manual screening of the reference lists of included sources. PubMed was selected for core biomedical indexing, Web of Science for broader interdisciplinary citation coverage, and ClinicalKey to enhance retrieval of the clinically oriented dental literature and practice-relevant publications. Scopus and Embase were not accessible through our institutional library subscriptions at the time the searches were conducted and therefore could not be included. This represents a practical access limitation rather than a deliberate exclusion of broader interdisciplinary sources. Because the review topic spans dentistry, artificial intelligence, informed consent, ethics, and data governance issues, the search strategy was designed to prioritize broad conceptual coverage.

The search strategy was developed around three core concept groups: (1) artificial intelligence, (2) dentistry/oral health, and (3) informed consent/ethical governance concepts. Controlled vocabulary and free-text terms were combined where appropriate and adapted to the indexing structure and search functionality of each database. The final database searches were conducted on 2 February 2026. The full database-specific search strings, keywords/concept blocks, limits, and retrieval counts are provided in Supplementary Material 2. Google Scholar was used only as a supplementary gray literature screening source after completion of the primary database searches. Search results were sorted by relevance, and the first 10 pages of results (100 records) were screened for each query string. This 10-page threshold was applied consistently across all query strings and constituted the prospectively applied stopping criterion for the Google Scholar component. Only records not previously identified through the primary database searches were assessed against the eligibility criteria. Because Google Scholar retrieval is dynamic, relevance-ranked, and personalization-sensitive, this component is inherently less reproducible than indexed database searches. Potentially relevant records were then assessed using the same eligibility criteria as indexed sources.

### 2.1. Eligibility Criteria

#### 2.1.1. Inclusion Criteria

Studies, reports, or documents were eligible for inclusion if they met all of the following criteria:

Population/Stakeholders: Dental patients, dental clinicians (e.g., general dentists, specialists, dental radiologists, oral surgeons), or participants in dental research; studies addressing patient–clinician–AI interactions in dentistry.

Concept: Informed Consent and AI: Explicit discussion of informed consent, patient information, autonomy, transparency, or shared decision-making in the context of AI; ethical, legal, or governance implications of AI use in clinical dentistry or dental research. Consent for Clinical AI Research (added explicitly): Articles addressing consent for AI research, including: secondary use of dental data (imaging, intraoral scans, photographs, salivary or behavioral data); consent for algorithm training or continuous learning systems; governance of AI research under GDPR, EU AI Act, WHO, or equivalent frameworks; IRB/ethics committee considerations for AI-based dental research.

Context: AI-Augmented Dentistry or Dental Research: AI applications in dentistry, including but not limited to: diagnostic imaging (e.g., OPT, CBCT, caries detection); clinical decision support systems (CDS); AI-assisted treatment planning; ambient AI scribes or automated clinical documentation; patient-facing AI tools (chatbots, mHealth, wearables); AI systems used for research purposes, including model development, validation, training, or secondary data use; settings encompassing clinical care, academic dentistry, or dental AI research environments.

Types of Sources: Peer-reviewed journal articles (original research, reviews, ethical analyses); legal or regulatory documents (e.g., GDPR, EU AI Act, policy statements); professional guidelines or position papers (e.g., WHO, FDI); relevant gray literature (white papers, authoritative reports).

Language and Timeframe: publications written in English, published from January 2015 to January 2026, reflecting contemporary AI applications in dentistry.

Because the review question addressed informed consent across clinical, ethical, legal, and governance dimensions of AI in dentistry, eligible sources were intentionally heterogeneous and included empirical studies, reviews, ethical analyses, professional guidance, regulatory/legal documents, and selected gray literature.

#### 2.1.2. Exclusion Criteria

Studies or documents were excluded if they met any of the following criteria: lack of relevance to dentistry; AI studies conducted exclusively in medicine or healthcare without dental applicability; engineering or computer science papers with no discussion of clinical, ethical, or consent implications; absence of an informed consent or ethical dimension; articles describing AI performance, accuracy, or technical development without reference to: informed consent, patient communication, ethical, legal, or governance considerations; studies focused solely on algorithm architecture, training performance, or validation metrics; AI benchmarking or simulation studies without patient involvement or ethical discussion; AI applications unrelated to clinical dental care or dental research (e.g., administrative AI without patient interaction); educational AI tools without patient data or consent implications; opinion pieces without ethical analysis; commentaries, or opinion articles lacking substantive ethical, legal, or conceptual analysis of informed consent; full texts unavailable for review. Robotic surgical systems performing autonomous operative procedures without a primary focus on informed consent in dental AI-assisted decision-making were excluded, as they represent a distinct technological and ethical domain beyond the scope of this review.

All references identified through the database searches were imported into Zotero reference management software (Zotero, Corporation for Digital Scholarship, Vienna, VA, USA), and duplicate records were identified and removed prior to the screening process. Before formal screening, the two primary reviewers (T.M. and C.M.C.) undertook a calibration exercise using a pilot sample of 30 randomly selected records (approximately 10% of the post-deduplication set), drawn from all three primary databases to ensure representativeness. Each reviewer independently applied the eligibility criteria to all 30 records and subsequently compared decisions. Initial agreement on the pilot sample was 87% (26/30 records). The four discordant records were discussed until consensus was achieved, and these discussions led to minor clarification of how the eligibility criteria should be applied to borderline cases, specifically regulatory documents without dental-specific content and AI performance papers containing only a brief ethics discussion. These clarifications were documented and applied consistently throughout the remainder of the screening process. A formal inter-rater agreement statistic (e.g., Cohen’s kappa) was not pre-specified in the review protocol and was therefore not calculated.

Titles and abstracts were then screened independently for relevance by the same two reviewers using the Rayyan platform (https://www.rayyan.ai/; accessed on 28 January 2026). Full-text records considered potentially eligible were subsequently reviewed independently by the same reviewers. Disagreements at either stage were resolved through discussion and consensus, with consultation of a third reviewer (V.N.) when necessary. Any minor clarifications in the operational application of the eligibility criteria remained consistent with the predefined Population–Concept–Context framework and review question and were applied uniformly throughout the remainder of the review.

Data were charted using a clinician-oriented extraction framework designed to capture consent elements directly relevant to dental practice, including disclosure of AI involvement, clinician oversight, AI-specific risks, data governance, and distinctions between clinical and research consent.

During data charting, each included record was classified by source category (peer-reviewed empirical study, systematic or scoping review, ethical or conceptual analysis, regulatory or legal document, clinical guideline or position paper, or gray literature) and this classification was used descriptively during synthesis. In thematic mapping, it informed interpretation of the evidentiary character of each theme: themes supported mainly by empirical records were described as observationally or survey-based, whereas those supported predominantly by ethical analyses, regulatory documents, or expert opinion were characterized as conceptual or normative. No formal hierarchy or weighting was applied across categories, consistent with scoping review methodology. Gray literature records were extracted using the same framework as indexed sources and contributed to the thematic synthesis on the same basis. A formal risk-of-bias assessment was not performed because this scoping review aimed to map a heterogeneous body of empirical and non-empirical literature rather than to synthesize intervention effects or produce a quality-weighted estimate of evidence.

The PRISMA-ScR checklist and the detailed search strategies for the main databases (PubMed, Web of Science, and ClinicalKey) are provided in the Supplementary Materials.

## 3. Results

**Figure 2:**
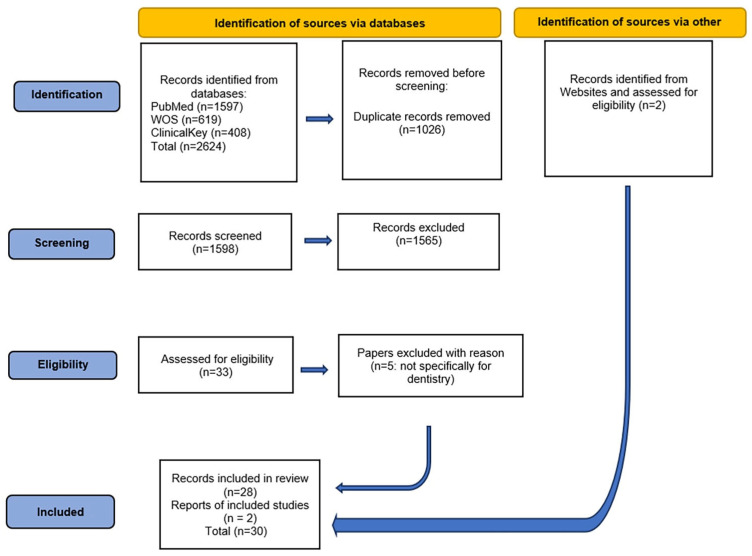
Adapted PRISMA 2020-style flow diagram illustrating the study selection process for this scoping review, reported in accordance with PRISMA-ScR.

A total of 2624 articles were initially identified through searches of three primary databases, and two additional eligible articles were retrieved from Google Scholar. As illustrated in Figure 2, after the removal of duplicates and completion of title/abstract screening and full-text eligibility assessment, 28 records from the primary databases and two gray literature records identified through Google Scholar were included, resulting in a total of 30 records [4,5,15,16,19,23,32,33,34,35,36,37,38,39,40,41,42,43,44,45,46,47,48,49,50,51,52,53,54,55]. Following a pilot calibration exercise on 30 records, title/abstract and full-text screening proceeded independently. Disagreements were infrequent and were resolved by discussion, with third-reviewer mediation when required. A list of full-text records excluded after eligibility assessment, together with reasons for exclusion, is provided in Supplementary Material 3, while the 30 included sources and their key characteristics are listed in Supplementary Material 4.

Table 1 presents a clinician-oriented synthesis of the essential informed consent elements reported in the included literature on AI-augmented dentistry. The table organizes evidence across key domains relevant to dental practice, including disclosure of AI involvement, professional responsibility, AI-specific risks, consent structure, and patient understanding. This synthesis provides a practical overview of recurring consent-related themes and recommendations described in the included sources, which may inform the development and implementation of AI-adapted consent approaches in dental settings.

To further contextualize these findings, Table 2 maps consent requirements according to the clinical, research, and hybrid contexts in which AI systems are deployed in dentistry.

The mapped literature revealed several recurring gaps, including limited empirical evidence, inconsistent differentiation between clinical and research consent, variable discussion of clinician accountability and AI-related risks, and a lack of standardized consent-oriented frameworks tailored to dental AI. Based on the thematic synthesis of included records, we propose the ACCOUNT-AI framework as an author-derived conceptual synthesis of recurring domains relevant to informed consent in AI-augmented dentistry (Table 3).

The framework is organized into seven structured domains, designed to be directly translatable into consent documentation and clinical workflows.

**Table 3:** Mapping of recurring synthesis themes to the ACCOUNT-AI framework domains and proposed consent elements for AI-augmented dentistry.

ACCOUNT-AI Domain	Recurring Theme(s) Identified in the Synthesis	Proposed Patient Disclosure/Consent Elements	Purpose and Ethical Justification	Predominant Source Type(s)	Representative References
**A** **—AI Role Clarification (Functional Transparency)**	Disclosure of AI involvement in diagnosis, treatment planning, documentation, or risk prediction; clarification of whether outputs are assistive, probabilistic, deterministic, validated, experimental, or adaptive	Inform patients whether AI is used, the clinical or research function it serves, and the nature of its outputs and validation status	Addresses hidden automation, supports meaningful understanding, and reduces opacity in clinical reasoning	Ethical analyses, reviews, regulatory/guidance sources	[23,40,54]
**C—Clinician Accountability and Oversight**	Dentist retains ultimate responsibility for decisions; AI outputs require human review and may be overridden; clinician competence is necessary for explanation and safe use	Clarify that the dentist remains responsible for all clinical decisions and that AI functions as a decision support tool rather than an autonomous decision-maker	Reinforces professional accountability, preserves legal and ethical responsibility, and mitigates unsafe delegation	Ethical analyses, reviews, survey-based empirical studies	[16,33,40,41]
**C—Context Differentiation (Care vs. Research vs. Hybrid Use)**	Distinction between AI use in direct patient care, AI-based research, and hybrid or continuously learning systems; need for separate or tiered consent pathways	Distinguish AI use for clinical care, research, and hybrid applications, and clarify when separate consent pathways are required	Prevents conflation of treatment with experimentation and supports context-appropriate consent	Ethical analyses, legal/regulatory sources, reviews	[23,25,40,55]
**O—Operational Risks and Limitations**	Disclosure of false positives/false negatives, algorithmic bias, explainability limits, automation bias, and performance variability across contexts or populations	Explain AI-specific risks, uncertainties, and limitations relevant to patient decision-making	Aligns AI-related disclosure with conventional risk communication and supports informed choice	Ethical analyses, reviews, some survey-based empirical sources	[16,32,38,54]
**U—Use and Reuse of Data (Secondary Data Governance)**	Secondary use of clinical data for AI development or validation; anonymization/pseudonymization; data sharing across institutions; opt-in/opt-out structures	Clarify whether patient data may be reused for AI development, how they are protected, whether they may be shared, and whether patients can opt in or opt out	Addresses privacy, autonomy, and governance concerns linked to secondary data use	Legal/regulatory analyses, governance reviews, white papers	[40,55]
**N—Navigable and Adaptive Consent Structure**	Tiered, layered, and dynamic consent models; AI-specific acknowledgment; proportionality between degree of AI involvement and disclosure burden	Use structured and adaptable consent formats, including layered explanations and AI-specific acknowledgment where appropriate	Supports proportional, comprehensible, and practicable consent processes	Ethical analyses, conceptual literature, commentary	[23,52,55]
**T—Transparency Across the AI Lifecycle**	Ongoing disclosure across development, validation, deployment, recalibration, continuous learning, and governance oversight	Integrate transparency not only at the point of clinical use, but across the AI lifecycle, including data reuse and system updating	Extends consent beyond one-time disclosure and frames transparency as a continuous ethical obligation	Ethical analyses, regulatory/guidance sources, reviews	[23,40,55]

This table integrates the conceptual content of the ACCOUNT-AI framework with its derivation from recurring themes identified in Table 1 and Table 2. Source types are presented descriptively to indicate the predominant nature of the evidence underpinning each domain and were not used hierarchically.

The ACCOUNT-AI framework proposed is grounded in a triadic dentist–patient–AI structure, with human oversight as the normative center. AI operates within clearly defined accountability boundaries, while transparency across the AI lifecycle ensures that patients are informed not only about the system’s role in decision-making but also about its development, validation, data reuse, and potential continuous learning.

Within this structure, secondary data reuse functions as a regulated feedback loop: responsibly governed reuse of clinical data enables algorithm validation, recalibration, bias mitigation, and population representativeness, thereby enhancing diagnostic accuracy and safety over time. Transparency regarding this process transforms data reuse from a purely privacy concern into an ethically justified mechanism for improving clinical reliability and decision quality.

Thus, accountability anchors decision-making, transparency safeguards autonomy, and structured data stewardship supports the iterative refinement of AI systems within ethically defined limits.

## 4. Discussion

This scoping review mapped a persistent gap between the rapid integration of artificial intelligence into dental practice and the development of clearly operationalized informed consent approaches in the literature. While the ethical necessity of informed consent for AI-augmented dentistry is widely acknowledged across the literature [23,33,37,40,52,54], most publications remain conceptual, offering high-level ethical principles without providing concrete guidance for clinical implementation. Beyond transparency and data protection alone, the mapped literature also points to broader ethical and legal concerns relating to clinician accountability, validity of consent, secondary data governance, and the boundary between clinical care and research [36].

The evidence mapped in this review indicates that informed consent in AI-augmented dentistry is currently fragmented, variably interpreted, and often inadequately adapted to the unique characteristics of AI systems. In particular, the literature highlights deficiencies in standardized terminology, clarity regarding clinician responsibility, differentiation between clinical care and research consent, and communication of AI limitations and uncertainty [40,41,52,54,56]. These shortcomings risk reducing consent to a formalistic exercise rather than a meaningful process that supports patient understanding and trust.

### 4.1. Informed Consent Beyond Disclosure: From Ethical Principles to Clinical Practice

A central finding of this review is that informed consent for AI in dentistry cannot be reduced to simple disclosure of AI use. A notable divergence exists between sources that treat disclosure as primarily a procedural obligation—satisfied by informing patients that AI is involved—and those that adopt a substantive model requiring communication of the AI system’s outputs, limitations, validation status, and potential for bias [23,54]. Roganović [23] and Rokhshad et al. [54] represent this more substantive position, whereas many of the broader reviews [5,8,13,25] acknowledge the need for disclosure without specifying its content. This distinction is clinically significant: procedural disclosure may satisfy a legal minimum while failing to support genuine patient autonomy, particularly when AI systems produce probabilistic or explainability-limited outputs. Traditional consent models, developed for stable, human-driven clinical interventions, are poorly suited to AI systems that may operate probabilistically, evolve over time, and rely on large-scale data processing [23,40,51,55]. Although many sources—predominantly conceptual analyses, ethical frameworks, and regulatory documents—emphasize the ethical obligation to inform patients when AI is involved in their care, few specify what information is essential, how it should be communicated, or how clinician oversight should be documented in practice. More broadly, recent reviews also frame these challenges in terms of implementation barriers, ethical integration, and the need to align consent processes with the expanding scope of AI use across dentistry [15,43,44,46].

Across the included literature, there is broad normative agreement that consent processes should explicitly address the AI system’s role in clinical reasoning, the nature of its outputs, and its limitations, including potential bias and uncertainty [16,23,40,57]. Importantly, the literature converges on the principle that dentists must retain ultimate responsibility for AI-assisted decisions, even when AI systems significantly influence diagnostic or treatment recommendations [33,40]. However, how this responsibility is exercised, communicated, and recorded remains insufficiently defined, creating ambiguity for both clinicians and patients.

### 4.2. Clinical Care Versus Research: A Persistent Ethical Fault Line

One of the most significant challenges identified in this review is the blurred boundary between AI-assisted clinical care and AI-based research. While traditional ethical frameworks draw a clear distinction between treatment and research, AI systems frequently occupy a hybrid space, particularly when clinical data are reused for algorithm training, validation, or continuous improvement [52,55]. The literature—largely consisting of ethical analyses and regulatory commentaries rather than empirical studies—consistently emphasizes that consent for clinical care does not automatically authorize secondary data use for AI development and that separate or tiered consent mechanisms are ethically preferable [23,25,55]. This concern also resonates with the broader medico-legal literature on informed consent in dentistry, which underscores that valid consent depends on context-specific disclosure, legal clarity, and meaningful patient understanding rather than on generic authorization alone [19].

Despite this consensus, practical solutions for managing dual-purpose data use in dental practice remain underdeveloped. Regulatory frameworks such as GDPR and the EU AI Act [40] impose transparency and consent requirements but offer limited dental-specific guidance on how to operationalize these obligations [4,47]. As a result, dentists may unknowingly rely on inadequate consent processes, exposing patients to unrecognized data uses and undermining trust in AI-assisted care. A critical tension is evident between sources that treat the care–research boundary as clearly definable [23,55] and those that implicitly acknowledge its blurriness in practice without providing resolution [4,40]. Brinz et al. [55] provide the most operationally detailed treatment of secondary data governance—including GDPR compliance, anonymization, and opt-in structures—but focus primarily on the researcher’s perspective. In contrast, Roganović [23] approaches the same boundary from the clinician–patient consent interaction. Neither source addresses how the boundary should be navigated in real-time during patient consultations where clinical and research AI uses may overlap, reflecting a genuine gap rather than a resolved consensus.

### 4.3. Emerging Consent Models: Promise Without Validation

The review identifies growing interest in adaptive consent models—particularly tiered, layered, dynamic, and risk-based approaches—as potential solutions to the limitations of traditional consent frameworks [23,42,58]. These models aim to tailor consent requirements to the level of AI involvement and risk, thereby balancing patient autonomy with clinical practicality. Tiered consent allows patients to choose different levels of AI participation or data sharing, while layered consent seeks to improve comprehension by presenting information progressively [23,52].

Dynamic consent, enabled by digital platforms, offers ongoing patient control over data use and consent preferences, which is particularly appealing for continuously learning AI systems [58].

While tiered and dynamic consent models are consistently described as promising [23,52,58], a critical reading of the literature reveals that their appeal rests heavily on theoretical logic rather than tested feasibility. Roganović [23] and Rokhshad et al. [54] both recommend layered or tiered approaches, but neither addresses implementation burden—including the time cost for dentists, the literacy demands on patients, or the infrastructure required for digital dynamic consent platforms. The single empirical test of a structured consent tool in this review (Roganović [23], pilot survey, n = 12 dentists) reported high usability ratings, but the sample is too small and clinician-only to support strong conclusions about patient acceptance or real-world uptake. The enthusiasm for adaptive models in the literature thus outpaces the evidence supporting them, which the review maps as a convergent normative preference rather than an evidence-based recommendation.

The evidence base supporting these approaches in dentistry is largely theoretical and normative. No empirical studies were identified in this review that evaluated the feasibility, patient comprehension, clinician workload impact, or long-term trust outcomes of tiered, dynamic, or layered consent models in dental AI settings. This reflects a mapped absence of empirical evidence, not a conclusion that such effects do not exist. As such, while these models are conceptually attractive, their real-world applicability in busy dental practices remains uncertain.

### 4.4. Patient Understanding, Trust, and the Dentist–Patient–AI Relationship

Patient understanding and trust emerge as central, yet underexplored, dimensions of informed consent in AI-augmented dentistry. A small number of conceptual and survey-based sources suggest that transparency about AI use can strengthen trust when accompanied by clear clinician explanation and reassurance of human oversight. Conversely, inadequate communication or overreliance on AI may erode the dentist–patient relationship, particularly if patients perceive AI as replacing human judgment. These issues are not limited to general dentistry and have also been noted in specialty contexts such as prosthodontics and implant dentistry, pediatric and special care dentistry, oral oncology, and dental public health, where AI may influence communication, expectations, and ethical risk in different ways [39,45,49,50].

Moreover, despite the expanding literature regarding AI’s applications in dentistry, just a few authors emphasize the necessity of disclosing potential conflicts of interest to ensure transparency and maintain patient trust in artificial intelligence (AI) [33,35]. However, empirical studies evaluating the integration of such disclosures into AI-related informed consent processes were not identified. This mapping outcome suggests an important gap in the literature, with potentially relevant ethical and legal implications for patient autonomy and the integrity of AI-assisted clinical decision-making.

Clinician uncertainty about AI systems has been identified in survey-based studies as directly affecting the quality of consent communication [16,23]. These findings, while based on a small number of survey studies, represent the primary empirical grounding for this theme [41].

A methodological limitation across the empirical studies in this corpus is that patient perspectives are largely absent. Of the seven sources with empirical data, six focus on clinician attitudes or expert consensus (Roganović [23,52], El Khoury et al. [35], Rokhshad et al. [42,54], Vaira et al. [53]). The sole source directly evaluating patient-facing AI output quality—Vaira et al. [53]—assesses the readability of AI-generated consent documents by oral surgeons, not by patients. This systematic clinician centrism in the empirical literature means that conclusions about what patients understand, need, or prefer in the context of AI-augmented consent remain speculative rather than evidence-based. The review maps this as a structural gap rather than an incidental absence.

### 4.5. AI-Generated Consent Documents: A Recursive Ethical Challenge

An emerging and particularly complex issue identified in this review is the use of AI itself to generate or assist consent documents. Preliminary studies suggest that AI-generated consent materials may improve readability and completeness; however, empirical evidence regarding their safety, acceptability, and impact on patient understanding remains extremely limited [59,60]. The use of AI to explain AI introduces a recursive ethical challenge, raising questions about accuracy, accountability, and transparency.

The literature consistently cautions against uncritical reliance on AI-generated consent without human review and oversight [59]. From an ethical perspective, patients should be informed not only about AI use in their care but also about AI involvement in the consent process itself. This highlights the need for what has been described as higher-order or meta-level consent governance, in which patients can express preferences regarding how consent decisions are made and managed over time [34].

### 4.6. Implications for Dental Practice and Policy

Taken together, the findings of this review suggest that informed consent for AI-augmented dentistry must evolve from a static, document-centered process to a dynamic, relationship-centered practice. Dentists should be supported by clear professional guidelines, standardized consent elements, and practical communication tools that reflect the realities of AI-assisted care. Regulatory frameworks provide an important foundation, but dental-specific implementation guidance remains urgently needed. In practical terms, this means that ethical decision-making in AI-assisted dentistry cannot rely on transparency alone, but also requires clinically meaningful communication, clearly defined professional responsibility, and proportionate governance of data use across care and research settings.

From a policy perspective, the absence of standardized consent frameworks risks variability in practice, legal uncertainty, and erosion of patient trust. The literature also suggests that specialty-specific adoption, broader system-level integration, and workforce preparedness should be considered when translating AI-related consent principles into routine dental practice [5,48]. The development of consensus-based consent models, informed by empirical research and aligned with evolving regulations, represents a critical next step for the dental profession.

The ACCOUNT-AI framework proposed in this review is an author-derived conceptual synthesis that responds to fragmentation identified in the literature by organizing recurring ethical and consent-related themes into structured domains relevant to dental practice and research. By integrating AI role clarification, clinician accountability, contextual differentiation, operational risk disclosure, secondary data governance, adaptive consent design, and lifecycle transparency, the framework provides a coherent structure capable of accommodating both current AI applications and emerging continuous learning systems. Importantly, it reframes secondary data reuse not solely as a privacy concern but as a governance-regulated mechanism for improving algorithmic calibration, bias mitigation, and diagnostic accuracy. In doing so, the framework seeks to align patient autonomy with collective clinical benefit while preserving the centrality of human professional responsibility within the dentist–patient–AI relationship. The proposed framework has not yet been empirically validated and should be understood as a synthesis-based proposal intended to support future refinement, empirical testing, and implementation research.

In practical terms, this suggests that dental consent processes involving AI should include, at minimum, explicit disclosure of AI involvement, clarification of clinician oversight, communication of relevant limitations and uncertainties, and clear explanation of any secondary data use where applicable. Pending empirical validation of specific consent models, these elements may serve as a pragmatic starting point for ethically robust disclosure and documentation in AI-augmented dental care.

### 4.7. Human Accountability in AI-Augmented Dental Care: Implications for Informed Consent

A late 1970s statement attributed to IBM—“A computer can never be held accountable, therefore a computer must never make a management decision”—remains strikingly relevant in the era of artificial intelligence in healthcare. While contemporary AI systems far exceed the computational capabilities of earlier technologies, the ethical premise underlying this statement persists: AI systems cannot bear moral, professional, or legal responsibility for clinical decisions.

**Figure 3:**
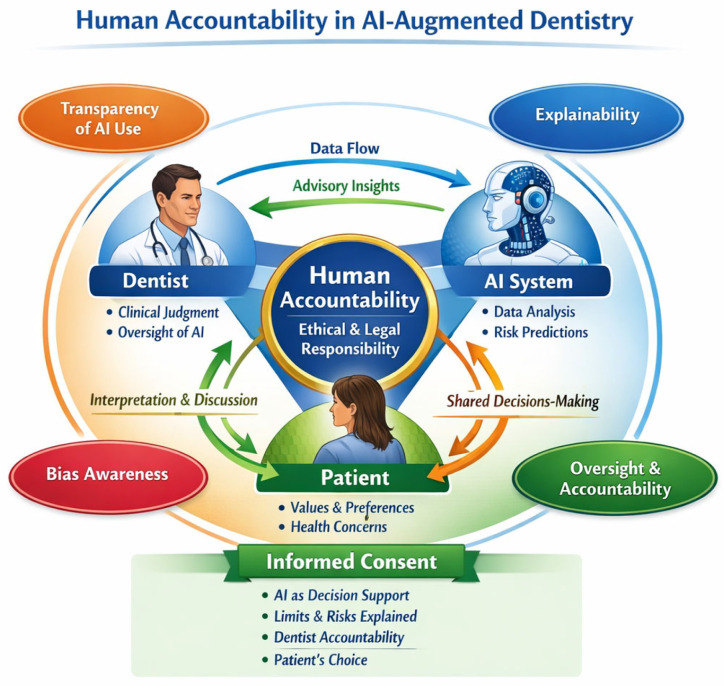
Human accountability in AI-augmented dental care: Implications for informed consent.

In AI-augmented dentistry, algorithms may analyze radiographs, predict caries risk, recommend orthodontic treatment plans, or stratify patients for implant success. However, AI outputs remain advisory tools rather than autonomous decision-makers. Accountability for diagnosis, treatment planning, and patient outcomes remains with the licensed dental professional (Figure 3). This distinction is not merely technical but foundational for informed consent.

### 4.8. Review Limitations

This scoping study is limited by the restriction to English-language publications and to the period January 2015 to January 2026. The timeframe was selected to focus on contemporary AI applications in dentistry and their associated ethical, legal, and governance implications. The English-language restriction was applied for feasibility and because much of the internationally influential regulatory and publication ethics literature relevant to AI in healthcare is disseminated in English. However, this approach may have excluded region-specific and multilingual scholarship, including local ethics, legal, regulatory, and professional guidance that may be relevant to informed consent in AI-augmented dental care and research. Additional limitations include the predominance of conceptual rather than empirical sources and the inherent methodological constraints of scoping reviews, which map existing evidence but do not formally assess study quality or provide quantitative synthesis.

Consistent with scoping review methodology, no formal risk-of-bias or quality assessment was applied to the included sources. While this is methodologically appropriate for a review that aims to map breadth of evidence rather than synthesize intervention effects, it has substantive implications for the robustness of the findings. The corpus is dominated by unsystematic reviews, ethical analyses, and expert commentaries, which are inherently susceptible to confirmation bias, selective citation, and normative advocacy. The empirical sources included have small samples (e.g., Roganović [23], *n* = 12; El Khoury et al. [35], cross-sectional single-country; Roganović et al. [41], *n* = 193) and represent limited geographic and professional diversity. The Delphi and e-Delphi studies [42,54] provide a stronger methodological grounding for consensus claims, but their expert panels may not represent the full range of dental practice contexts globally. Taken together, these characteristics mean that the mapped themes represent the positions of a relatively concentrated scholarly network working on AI ethics in dentistry, and may not fully reflect practice realities, patient perspectives, or regulatory contexts in lower-resource or non-European settings. Future evidence synthesis in this field would benefit from systematic reviews with formal quality appraisal as the evidence base matures.

### 4.9. Future Directions

This review highlights several priorities for future research, including empirical studies on patient preferences and understanding, comparative evaluations of different consent models, and implementation research in real-world dental settings. Particular attention should be paid to vulnerable populations, health literacy, and equity, as AI systems and consent processes may differentially affect diverse patient groups.

Ultimately, informed consent in AI-augmented dentistry should be understood not merely as a regulatory obligation but as a core expression of patient-centered care. As AI becomes increasingly embedded in dental practice, the profession has both an ethical responsibility and a practical imperative to ensure that consent processes are meaningful, transparent, and responsive to the evolving dentist–patient–AI relationship.

Emerging fully autonomous robotic interventions may introduce additional consent complexities, but these fall outside the present review’s focus on AI-augmented clinical decision support and data-driven systems.

With respect to the ACCOUNT-AI framework specifically, a staged validation pathway is warranted. First, a Delphi consensus exercise involving dental clinicians, ethicists, patient representatives, and AI developers could be undertaken to assess the framework’s domains, content, and completeness, and to identify areas requiring addition, refinement, or greater specificity. Second, the framework should be piloted across diverse dental practice settings, including primary care, specialist, and academic contexts, in order to evaluate operational feasibility and identify implementation barriers. Third, patient-facing usability testing should examine whether the proposed consent elements support meaningful patient comprehension and autonomous decision-making across different levels of health literacy. Finally, longitudinal evaluation of framework-informed consent processes should assess outcomes such as patient satisfaction, trust, clinician confidence, and the validity of consent in relation to ethical and legal standards. Taken together, this stepwise pathway would support progression from a conceptual synthesis to an expert-informed, practice-tested, and patient-centered framework, consistent with established approaches to framework development and validation in health sciences research.

## 5. Conclusions

The conclusions of this scoping review are grounded in a body of literature that is predominantly conceptual, ethical, and narrative in nature. Of the 30 sources included, 23 (77%) are reviews, ethical analyses, position papers, or commentaries; seven (23%) include empirical data, of which none directly measures patient consent outcomes in AI-augmented dental practice. The findings therefore reflect a mapped normative consensus rather than an evidence-based consensus in the strictest sense, and should be interpreted accordingly. The identified themes and gaps represent what the scholarly and professional literature currently proposes and discusses, not what has been empirically demonstrated to improve or harm patient outcomes.

This scoping review mapped a persistent gap between the rapid integration of artificial intelligence into dental practice and the development of clearly articulated, context-sensitive approaches to informed consent. While transparency, clinician accountability, and respect for patient autonomy are consistently recognized as foundational principles, their practical translation into structured consent processes remains fragmented and inconsistent.

The mapped literature suggests that informed consent in AI-augmented dentistry may need to move beyond a traditional bilateral clinician–patient model toward a more explicit dentist–patient–AI framework grounded in human oversight and transparency across the AI lifecycle. In response to the conceptual heterogeneity identified in the literature, this review proposes the ACCOUNT-AI framework as an author-derived conceptual synthesis that organizes recurring consent-related themes into seven structured domains, encompassing AI role clarification, clinician accountability, contextual differentiation between care and research, AI-specific risk disclosure, secondary data governance, adaptive consent formats, and transparency across the AI lifecycle.

The included literature also suggests that transparent and ethically governed secondary data reuse may contribute to model refinement, bias mitigation, and improved clinical performance; however, the practical implications of these processes for consent remain insufficiently evaluated in dental settings. Empirical validation of consent models in real-world dental settings also remains limited. Future research should prioritize implementation studies, patient comprehension assessment, clinician education, and evaluation of dynamic consent mechanisms in order to test and refine consent approaches for AI-augmented dentistry and to strengthen their ethical and practical relevance.

## Figures and Tables

**Table 1:** Clinician-oriented synthesis of the recurring informed consent themes identified across the included sources on AI-augmented dentistry.

Domain	Key Consent Element	Information Commonly Recommended for Communication to Patients (Dentist/Oral Surgeon Perspective)	Clinical and Ethical Relevance	Key References
**AI Disclosure Practices**	Disclosure of AI involvement	The included sources commonly recommended informing patients when AI contributes to diagnosis, treatment planning, imaging interpretation, documentation, or decision support	Prevents hidden automation and supports informed decision-making	Roganović (2025) [23]; Rokhshad et al. (2023) [54]; Ducret et al. (2024) [40]
	Nature of AI output	Explain whether AI outputs are deterministic or probabilistic, and whether the system is validated or experimental	Sets realistic expectations and mitigates overreliance	Roganović (2025) [23]; Rokhshad et al. (2023) [54]
	Documentation of disclosure	Record AI disclosure in the clinical file, including AI role and clinician review	Supports medico-legal traceability	Roganović (2025) [52]
**Clinician Accountability & Oversight**	Final responsibility	The included sources consistently emphasized that the dentist retains ultimate clinical responsibility for decisions	Reinforces professional accountability and legal clarity	Sciarra et al. (2025) [16]; Ducret et al. (2024) [40]
	Human oversight	Confirm that AI outputs are reviewed and may be overridden by the clinician	Prevents automation bias and unsafe delegation	Weerakoon et al. (2025) [38]; Ducret et al. (2024) [40]
	Clinician competence	Ensure the clinician understands AI system limits and performance	Ethical obligation to avoid misuse of AI	Roganović and Radenković (2023) [33]; Weerakoon et al. (2025) [38]
**Clinical Care vs. Research Consent**	Separate consent pathways	Distinguish consent for AI-assisted clinical care from consent for AI research	Prevents ethical conflation of care and research	Roganović (2025) [23]; Brinz et al. (2025) [55]; Ducret et al. (2024) [40]
	Secondary data use	Several included sources recommended explicit opt-in consent for reuse of clinical data in AI training or validation	GDPR and research ethics compliance	Brinz et al. (2025) [55]; Ducret et al. (2024) [40]
	Data protection	Inform patients about anonymization, de-identification, and privacy safeguards	Addresses data governance concerns	Brinz et al. (2025) [55]; Ducret et al. (2024) [40]
**AI-Specific Risks**	Algorithmic bias	Explain that AI may perform differently across populations or clinical contexts	Supports fairness and risk awareness	Rokhshad et al. (2023) [54]; Ducret et al. (2024) [40]
	Diagnostic errors	Disclose risks of false positives/negatives and model limitations	Aligns AI risks with conventional clinical risk disclosure	Rahim et al. (2024) [32]; Rokhshad et al. (2023) [54]
	Explainability limits	Inform patients when AI decisions are not fully interpretable	Ethical transparency requirement	Ducret et al. (2024) [40]; Rahim et al. (2024) [32]
	Right to refuse AI	Some included sources discussed the availability of non-AI alternatives as relevant to preserving patient autonomy where feasible	Preserves patient autonomy	Roganović (2025) [52]; Rokhshad et al. (2023) [54]
**Consent Formats**	Structured AI consent elements	Include AI role, benefits, risks, clinician oversight, and data use	Standardizes AI disclosure across dental practice	Rokhshad et al. (2023) [54]; Roganović (2025) [23]
	Tiered/layered consent	Adapt depth of explanation to level of AI involvement and risk	Improves comprehension without overburdening patients	Rokhshad et al. (2023) [54]; Roganović (2025) [23]
	AI-specific acknowledgment	Use a separate checkbox or signature line for AI use	Makes AI consent explicit and auditable	Rokhshad et al. (2023) [54]; Roganović (2025) [23]
**Patient Understanding and Trust**	Communication quality	Clinician explanation strongly influences patient trust in AI	Maintains therapeutic alliance	Roganović et al. (2023) [41]; Weerakoon et al. (2025) [38]
	Clinician confidence	Dentist uncertainty about AI undermines patient understanding	Highlights need for professional training	Roganović et al. (2023) [41]; Weerakoon et al. (2025) [38]
	Monitoring understanding	Assess patient comprehension during early implementation	Moves beyond formalistic consent	Weerakoon et al. (2025) [38]; Roganović (2025) [23]
**AI-Generated Consent Documents**	Use of AI to draft consent	AI-generated consent may improve readability but lacks validation	Prevents uncritical reliance on AI-generated text	Vaira et al. (2025) [53]; Roganović (2025) [23]
	Mandatory human review	AI-drafted consent must be reviewed and approved by a clinician	Ensures ethical and legal accuracy	Vaira et al. (2025) [53]; Roganović (2025) [23]
	Data provenance	Protect patient data used in generating consent text	Prevents secondary misuse of sensitive data	Brinz et al. (2025) [55]

**Table 2:** Context-dependent consent requirements for AI use in dentistry.

AI Context	AI Role	Consent Focus	Consent Considerations Emphasized in the Literature	Key References
**Routine clinical care (low-risk AI)**	Administrative support, image enhancement, scheduling	Transparency	General disclosure of AI use was commonly described as sufficient, whereas separate written consent was not consistently considered necessary.	Rokhshad et al. (2023) [54]; Ducret et al. (2024) [40]
**Clinical decision support (moderate risk)**	Diagnostic suggestions, treatment planning assistance	Autonomy & oversight	Explicit disclosure of AI role, limitations, and clinician responsibility; inclusion in written consent.	Roganović (2025) [23]; Rokhshad et al. (2023) [54]
**High-impact clinical AI**	AI significantly influences diagnosis or treatment decisions	Risk & accountability	The literature commonly emphasized explicit and documented consent, together with explanation of AI uncertainty, bias, and possible alternatives, including discussion of the patient’s ability to decline AI use where feasible.	Roganović (2025) [23]; Ducret et al. (2024) [40]
**Hybrid care–research AI**	Deployed AI still undergoing validation or learning	Dual-purpose transparency	Disclosure of developmental status; separate explanation of care vs. research functions.	Roganović (2025) [23]; Brinz et al. (2025) [55]
**AI-based research**	Model training, validation, algorithm development	Research ethics	Separate research consent; purpose, data use, withdrawal rights, data sharing.	Brinz et al. (2025) [55]; Ducret et al. (2024) [40]
**Secondary data use**	Retrospective data reuse for AI improvement	Data governance	Explicit opt-in consent; explanation of anonymization and sharing.	Brinz et al. (2025) [55]; Roganović (2025) [23]
**Dynamic/learning AI systems**	Continuous model updating	Ongoing autonomy	Tiered or dynamic consent; possibility to modify preferences over time.	Brinz et al. (2025) [55]; Roganović (2025) [23]
**AI-generated consent tools**	AI assists consent drafting or explanation	Meta-consent	Mandatory human review; disclosure of AI-generated content.	Vaira et al. (2025) [53]; Roganović (2025) [23]

## Data Availability

No new data were created.

## Supplementary Materials

The following supporting information can be downloaded at: https://www.mdpi.com/article/10.3390/dj14060320/s1, Supplementary Material 1: Preferred Reporting Items for Systematic reviews and Meta-Analyses extension for Scoping Reviews (PRISMA-ScR) Checklist; Supplementary Material 2: Search strategy; Supplementary Material 3: Full-text articles excluded after eligibility assessment, together with reasons for exclusion; Supplementary Material 4: Included sources and key characteristics.

## Abbreviations

The following abbreviations are used in this manuscript:

AI	Artificial intelligence
CDS	Clinical decision support
NLP	Natural language processing
CBCT	Cone beam computed tomography
LLM	Large language model
EHR	Electronic health record
mHealth	Mobile health
OSF	Open Science Framework
PCC	Population–Concept–Context
GDPR	General Data Protection Regulation
EU	European Union
FDI	World Dental Federation
WHO	World Health Organization
IRB	Institutional review board
