# Supplementary material for: Informed Consent in AI-Augmented Dentistry and Dental Research: A Scoping Review
Source: Dent J (Basel). 2026 May 25;14(6):320. doi: 10.3390/dj14060320 (PMC13298974; doi:10.3390/dj14060320)
Supplement: Supplementary file 1 [file dentistry-14-00320-s001.zip › Supplementary material 2.pdf]

Supplementary Material 2 - Search Strategy

Database	Keywords/ MeSH terms	Results
PubMed/MEDLINE<br>Timeframe filter: January 2015 to January 2026<br>Language filter: English	Full expanded PubMed syntax (Search Details translation, confirmed 02.02.2026): (("Artificial Intelligence"[MeSH Terms] OR "Machine Learning"[MeSH Terms] OR ("artificial"[All Fields] AND "intelligence"[All Fields]) OR "artificial intelligence"[All Fields] OR ("machine"[All Fields] AND "learning"[All Fields]) OR "machine learning"[All Fields] OR ("deep"[All Fields] AND "learning"[All Fields]) OR "deep learning"[All Fields] OR "large language model*"[All Fields] OR "chatbot*"[All Fields] OR "ambient scribe"[All Fields]) AND ("Dentistry"[MeSH Terms] OR "Dental Care"[MeSH Terms] OR "Oral Health"[MeSH Terms] OR "dentistry"[All Fields] OR "dental"[All Fields] OR "oral"[All Fields]) AND ("Informed Consent"[MeSH Terms] OR "ethics, clinical"[MeSH	1597

	Terms] OR ("informed"[All Fields] AND "consent"[All Fields]) OR "informed consent"[All Fields] OR "ethical"[All Fields] OR "ethically"[All Fields] OR "ethics"[MeSH Terms] OR "ethics"[All Fields] OR "ethic"[All Fields] OR "trust"[MeSH Terms] OR "trust"[All Fields] OR "trusted"[All Fields] OR "trusting"[All Fields] OR "trusts"[All Fields] OR "accountable"[All Fields] OR "accountability"[All Fields] OR "transparency"[All Fields] OR "transparent"[All Fields] OR "shared decision making"[All Fields]) AND ("research"[MeSH Terms] OR "research"[All Fields] OR "researcher"[All Fields] OR "researchers"[All Fields] OR "researching"[All Fields] OR "secondary data use"[All Fields] OR "data governance"[All Fields] OR "AI research"[All Fields] OR "human subjects research"[All Fields] OR "irb"[All Fields] OR "GDPR"[All Fields])) AND (2015/1/1:2026/1/31[pdat]) AND (english[Filter])	

Web of Science Core Collection<br>Timeframe filter: January 2015 to January 2026<br>Language filter: English	TS=((artificial intelligence OR machine learning OR "clinical decision support" OR chatbot*<br>OR "ambient scribe")<br>AND (dentistry OR "dental care" OR "oral health")<br>AND ("informed consent" OR ethics OR trust OR autonomy)) English language	619
ClinicalKey (Elsevier)<br>Timeframe filter: January 2015 to January 2026<br>Language filter: English	Artificial intelligence AND dentistry AND informed consent (English language)	408
Google Scholar (grey literature supplement)<br>Timeframe filter: January 2015 to January 2026<br>Language filter: English	Query strings used: "artificial intelligence" AND "dentistry" AND "informed consent"; "AI dentistry ethics"; "dental AI patient autonomy"; "machine learning dental consent".	2 included (from 4 identified across query strings)

The search strategies are presented in fully reproducible, database-specific syntax to reflect differences in indexing and search functionality across platforms. For PubMed, the string presented below is the complete expanded syntax as generated and confirmed by PubMed's Search Details translation tool (Advanced Search interface), which shows the exact internal query executed by the system, including Automatic Term Mapping (ATM) expansions and field tag assignments. Syntax was adapted to the indexing structure of each database. All database searches were conducted on 02.02.2026. Retrieval counts correspond to the searches as conducted on that date and are consistent with the PRISMA-ScR flow diagram. Minor variation in result counts upon subsequent replication is expected due to continuous database updates, MeSH re-indexing, and record corrections — a recognized characteristic of living bibliographic databases.

The Google Scholar search was conducted after completion of the primary database searches, screening, and selection process (28 records included from main databases). It was used exclusively to identify additional records not captured by the primary searches. Records already included from the main databases were not reassessed. Search results were sorted by relevance (default). The first

10 pages of results (100 records) were screened per query string, as beyond this threshold results became markedly irrelevant to the review topic. This 10-page threshold was applied consistently across all query strings and constituted the prospectively applied stopping criterion. Only records not previously identified through the main database searches were assessed against the eligibility criteria. Google Scholar was used as a supplementary, sequential, non-systematic additive source; its dynamic and relevance-ranked retrieval prevents full reproducibility, which is a recognized limitation of this search component.
