# Supplementary material for: Informed Consent in AI-Augmented Dentistry and Dental Research: A Scoping Review
Source: Dent J (Basel). 2026 May 25;14(6):320. doi: 10.3390/dj14060320 (PMC13298974; doi:10.3390/dj14060320)
Supplement: Supplementary file 1 [file dentistry-14-00320-s001.zip › Supplementary material 3.pdf]

Supplementary material 3.

Full-text articles excluded after eligibility assessment, with reasons for exclusion

First author, year	Title	Journal (Source)/DOI	Reason for exclusion
Resnik DB et al., 2025	The ethics of using artificial intelligence in scientific research: new guidance needed for a new tool.	AI Ethics/10.1007/s43681-024-00493-8.	Did not address dentistry specifically
Hurley ME et al.,2024	Patient Consent and The Right to Notice and Explanation of AI Systems Used in Health Care.	Am J Bioeth./ 10.1080/15265161.2024.2399828	Did not address dentistry specifically
Park HJ., 2024	Patient perspectives on informed consent for medical AI: A web-based experiment.	Digit Health./ 10.1177/20552076241247938.	Did not address dentistry specifically
Palaniappan K et al., 2024	Global Regulatory Frameworks for the Use of Artificial Intelligence (AI) in the Healthcare Services Sector.	Healthcare (Basel)./ 10.3390/healthcare12050562.	Did not address dentistry specifically
Kerasidou A. J., 2021	Ethics of artificial intelligence in global health: Explainability, algorithmic bias and trust.	Oral Biol Craniofac Res./ 10.1016/j.jobcr.2021.09.004.	Did not address dentistry specifically
