# Supplementary material for: Informed Consent in AI-Augmented Dentistry and Dental Research: A Scoping Review
Source: Dent J (Basel). 2026 May 25;14(6):320. doi: 10.3390/dj14060320 (PMC13298974; doi:10.3390/dj14060320)
Supplement: Supplementary file 1 [file dentistry-14-00320-s001.zip › Supplementary material 4.pdf]

Supplementary Material 4: Included sources and key characteristics

No.	Included source	Year	Source type	Dental context/domain	Primary basis for inclusion
1	Roganović (1)	2025	Ethical analysis	General dentistry	Informed consent for AI in dentistry
2	Vaira et al.	2025	Comparative study	Oral surgery	AI-generated consent; patient information quality
3	Rokhshad et al.	2023	Study / Delphi study	General dentistry	Ethical framework; consent-relevant guidance
4	Brinz et al.	2025	Narrative review	Dental AI research / data governance	Secondary data use; privacy; legal/governance issues
5	Rahim et al.	2024	Review	General dentistry	Ethical implications of AI in dentistry
6	Roganović (2)	2025	Review and study	General dentistry	Consent checklist; consent structure
7	Roganović & Radenković	2023	Review / book chapter	General dentistry	Ethical analysis; professional responsibility
8	Batra & Reche	2023	Review	General dentistry	Broad AI in dentistry; ethical/consent relevance
9	El Khoury et al.	2025	Cross-sectional study	General dentistry	Ethical perceptions; implementation concerns
10	Navdeep Kaur et al.	2025	Review	General dentistry	Ethical responsibility; governance
11	Feng et al.	2025	Narrative review	Oral medicine	Ethical risks; professional implications
12	Weerakoon et al.	2025	Scoping review	Dentistry / healthcare interface	Ethical and implementation issues

No.	Included source	Year	Source type	Dental context/domain	Primary basis for inclusion
13	Liu et al.	2025	Review	General dentistry	Ethical considerations; barriers to implementation
14	Alfaraj et al.	2024	Review	Prosthodontics / implant dentistry	Ethical considerations in specialty use
15	Ducret et al.	2024	Review	General dentistry / regulation	Legal-regulatory guidance; trustworthy AI
16	Roganović et al.	2023	Survey / ethical analysis	General dentistry	Professional attitudes; responsible use
17	Rokhshad et al.	2024	Study / e-Delphi consensus	Prosthodontics / esthetic dentistry	Ethical challenges; professional oversight
18	Schwendicke et al.	2023	White paper	General dentistry	Policy/guidance; ethics and implementation
19	Rokhshad et al.	2024	Systematic review / meta-analysis	Pediatric dentistry	AI applications with ethical relevance
20	Otero et al.	2022	Review	Dentistry / medico-legal consent	Informed consent; legal perspective
21	Tuygunov et al.	2025	Review	General dentistry	Ethical/peril-oriented discussion; professional guidance
22	Bailey	2024	Ethical commentary	General dentistry	Ethical integration; professional readiness
23	Sciarra et al.	2025	Review	General dentistry	Clinical responsibility; accountability
24	Assiry et al.	2025	Scoping review	Pediatric / special care dentistry	Ethical considerations in specialty care
25	Dua et al.	2025	Review	General dentistry	Future roadmap; ethical and practical implications

No.	Included source	Year	Source type	Dental context/domain	Primary basis for inclusion
26	ITU-WHO Focus Group on AI for Health Final Report	2025	Position paper / report	Health governance relevant to dental AI	Ethics; regulation; standardization; governance context
27	Harte et al.	2025	Review	Dental education	Ethical/professional preparedness relevant to future practice
28	Vinay et al.	2025	Scoping review	Oral cancer / diagnostics	AI applications with ethical relevance
29	Bamashmous	2025	Review	Dental public health	Ethical considerations; future directions
30	Mörch et al.	2021	Scoping review	General dentistry	Ethics in dentistry; conceptual groundwork
